# Pediatric living-donor liver transplantation using right posterior segment grafts

**DOI:** 10.1186/s12876-021-01835-0

**Published:** 2021-06-06

**Authors:** Xiaoye Qu, Ping Wan, Mingxuan Feng, Bijun Qiu, Yi Luo, Tao Zhou, Jianjun Zhu, Dong Zhao, Guangxiang Gu, Jianjun Zhang, Qiang Xia

**Affiliations:** Department of Liver Surgery, Renji Hospital, School of Medicine, Shanghai Jiaotong University, No. 160 Pujian Road, Pudong New District, Shanghai, 200127 China

**Keywords:** Pediatric liver transplantation, Live donor, Right posterior segment graft

## Abstract

**Background:**

The right posterior segment (RPS) graft was introduced to overcome graft size discrepancy in living donor liver transplantation (LDLT). However, it was very rarely used in pediatric patients. Here we presented 4 pediatric LDLT cases receiving RPS graft between January 2015 and April 2020 in our center. A total of 1868 LDLT procedures were performed in this period.

**Methods:**

Recipients included 1 boy and 3 girls with a median age of 45 months (range from 40 to 93 months). They were diagnosed with progressive familial intrahepatic cholestasis, propionic academia, ornithine transcarbamylase and biliary atresia, respectively. Four donors were all mothers with a median age of 32.5 years (31–38 years). Computer tomography angiography indicated posterior right branches branched off separately from main portal veins (type III variation). Three of these donor livers had 1 orifice of right hepatic veins (RHV). In the remaining 1 donor liver, the RHV showed 3 orifices and an outflow patch plastic was performed. Inferior right hepatic veins weren’t found in four donor grafts. The median graft weight was 397.5 g (352–461 g) and the median graft-to-recipient weight ratio was 2.38% (1.44–2.80%).

**Results:**

Postoperative complications occurred in neither donors nor recipients. Within the median follow-up duration of 29 months (14–64 months), four children are all alive with normal liver function.

**Conclusion:**

In summary, for older children weighed more than 15 kg with donors’ variation of type III portal veins, the use of RPS grafts could be a feasible and favorable option.

## Background

Living donor liver transplantation (LDLT) is widely performed for the treatment of end-stage liver diseases to overcome organ shortage [1]. Simultaneously minimizing the operative risks to donors and providing sufficient functional livers for recipients have become a great dilemma. Accurate preoperative evaluation, including volumetric and anatomical evaluation, plays an essential role in the success rate of this operation [2]. Selection of graft volume is the cornerstone. In pediatric liver transplantation, the left lateral lobe graft is most commonly used and fulfills major young patients’ requirements. Conversely, in some older children with higher weight, the left lateral lobe graft is usually insufficient for recipients’ liver function [3].

Comparing to the left lobe graft, the RPS graft may carry a higher risk to donors by the reason of more surgical difficulties [4]. In addition, suitable candidates as donors meeting anatomical requirements are relatively scarce. According to previous reports [5], the most favorable configuration occurs when the right posterior portal vein is branching separately from the main portal vein, which accounts for about 20.3 percent of healthy adults. Up to now, a few centers have reported the use of RPS graft in adult-to-adult LDLT [6], but rare cases in pediatric patients have been displayed. Here, we present our four pediatric LDLT cases using RPS graft from January 2015 to April 2020 at the Department of Liver Surgery, Renji Hospital.

## Methods

### Patients and characteristics

Between January 2015 and April 2020, 1868 pediatric patients underwent LDLT at the Department of Liver Surgery, Renji Hospital, School of Medicine, Shanghai Jiao Tong University. RPS grafts were used for 4 of these recipients. We retrospectively analyzed the clinical data and postoperative outcomes of these 4 cases. This study was approved by the institutional review board of Renji Hospital. Written informed consent was obtained from the patients for publication of this research and any accompanying images. The graft selection criterion has been strictly modified in our center. In general, we used the left lateral lobe as our first choice in pediatric LDLT because the left lateral lobe usually could provide sufficient liver volume in a child with the minimized donor risk. The RPS graft would be taken into consideration if the right posterior branch of PV branched off separately from the main portal vein (Type-III PV). The Existence and the domination area of inferior right hepatic veins (IRHV) would be strictly identified during imageological examination to evaluate the demand and difficulty in reconstructions of IRHV. Three-dimensional computed tomography (CT) was applied for the preoperative anatomical measurement and visualization of the donors’ vascular anatomy. The IQQA®-3D Liver system (EDDA TECHNOLOGY, USA) was applied to finish our volumetric calculation and analyses. In addition, Magnetic Resonance Cholangiopancreatography (MRCP) was conducted before the surgery to estimate the biliary tract. Intraoperative Cholangiography (IOC) was also performed during the operation to further conform the BD condition.

### Operative procedures

The surgery of recipients and donors were performed simultaneously. For RPS graft procurement, hilar dissection was carefully performed to identify vessels of the RPS. We then temporarily clamped these vessels including the right posterior branch of PV and HA. A boundary line between right anterior lobe and RPS would be clearly exposed and the parenchymal dissection was next performed along the ischemic demarcation. The Cavitron ultrasonic surgical aspirator (CUSA 200 system, Valleylab, Boulder, CO) was applied in this dissection process. During the parenchymal transection, all vascular branches on the dissection plane were cautiously isolated and ligated. The PV catheterization with University of Wisconsin solution (UW solution) perfusion was immediately performed after the graft procurement. During the implantation process of the liver, 5-0 and 6-0 PDS sutures were respectively used for continuous suturing of the PV reconstruction and outflow tract reconstruction. Anastomoses between the right hepatic artery of the recipient and the right posterior hepatic artery of the donor were completed under a surgical microscope with 8-0 Prolene sutures. End-to-end anastomosis was used for the bile duct reconstruction with 7-0 Maxson sutures.

### Postoperative management

For donors, the functions of remnant livers were tested every day after grafts procurement until the parameters had returned to normal. As for recipients, initial immunosuppressive therapy consisted of a dual drug regimen of tacrolimus combined with methylprednisolone. Doppler ultrasound (US) tests estimating the vascular system were performed daily in the first week after LT, every 2 days in the second week, monthly during the first 6 months, and every 3 months thereafter [7].

## Results

### Preoperative evaluation of recipients and donors

**Fig. 1:**
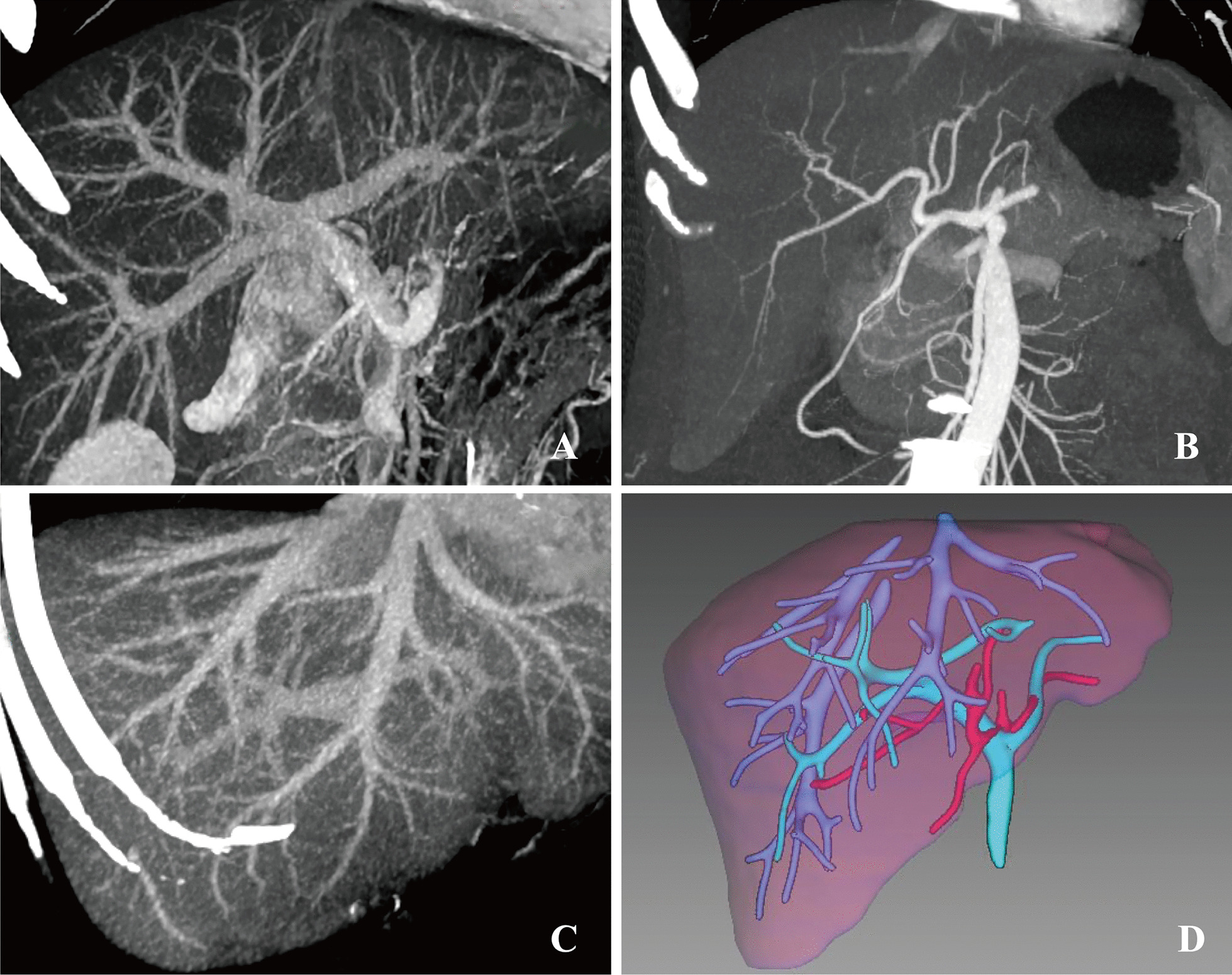
Donor’s preoperative CT 3-dimensional reconstruction of anatomical variations of case 1. **A** The Posterior right branch branched off separately from the main portal vein, which was classified as Type-III portal vein. **B** The right posterior hepatic artery branched extrahepatically. **C** The outflow system. **D** Three-dimensional imaging of hepatic blood vessels

Preoperative profiles of 4 children using RPS grafts and their donors were shown in Table 1. Recipients included 1 boy and 3 girls with a median age of 45 months (range from 40 to 93 months). They were diagnosed with progressive familial intrahepatic cholestasis (PFIC), propionic academia (PA), ornithine transcarbamylase (OTC) and biliary atresia (BA), respectively. One girl underwent Kasai procedure one month after her birth, while the other three children accepted no previous surgery. Four donors were all mothers with a median age of 32.5 years (31–38 years). The median weight was 53 kg (52–56 kg) and the median BMI was 20.4 kg/m^2^ (19.7–21.3 kg/m^2^). Each pair of the recipient and donor had identical ABO group. The detailed anatomical variations of donors were displayed in Table 2. PV, RHA, and RHD were classified using criterion addressed by Ulsan University College of Medicine [8]. All four donors showed Type- III portal veins, which indicated that posterior right branches branched off separately from the main portal veins. Right posterior hepatic arteries of all cases branched extrahepatically (Fig. 1). All right posterior bile ducts drained into the common hepatic ducts separately, which were classified as Type A. Three of these donor livers had 1 orifice of right hepatic veins (RHV). In the remaining 1 donor liver, the RHV showed 3 orifices and an outflow patch plastic was performed. Inferior right hepatic veins (IRHV) weren’t found in our cases and reconstructions between IRHV and RHV weren’t needed. The results of preoperative volumetric evaluation calculated by IQQA image analysis were also displayed in Table 2.

**Table 1:** Profiles of recipients and donors

Variable	Case 1	Case 2	Case 3	Case 4
Recipients				
Sex	Female	Male	Female	Female
Age at transplant(months)	93	49	41	40
Body height(cm)	120	102	89	96
Body weight(kg)	28.1	16	16.5	15.2
Diagnosis	PFIC	PA	OTC	BA
Previous surgery	–	–	–	Kasai procedure
Child–Pugh Score	B	A	A	C
ABO group	O	A	B	B
Donors				
Sex	Female	Female	Female	Female
Age at surgery	38	32	33	31
Body height(cm)	162	159	162	164
Body weight(kg)	53	52	56	53
BMI (kg/m^2^)	20.2	20.6	21.3	19.7
Relationship	Mother	Mother	Mother	Mother
ABO group	O	A	B	B

**Table2:** Preoperative evaluation of RPS donors

Variable	Case 1	Case 2	Case 3	Case 4
Anatomical variations				
PV	Type-III	Type-III	Type-III	Type-III
HA	Extrahepatic	Extrahepatic	Extrahepatic	Extrahepatic
BD	Type-A	Type-A	Type-A	Type-A
RHV orifice	3	1	1	1
Volumetric evaluation				
LLS (cm^3^)/GRWR (%)	146/0.52	148/0.93	185/1.12	134/0.88
LL with MHV (cm^3^)/GRWR (%)	360/1.28	312/1.95	398/2.41	295/1.94
RPS (cm^3^)/GRWR (%)	461/1.64	455/2.84	489/2.96	384/2.53

*PV* portal vein, *HA* hepatic artery, *BD* bile duct, *RHV* right hepatic vein

### Intraoperative findings and reconstructions during LDLT

**Fig. 2:**
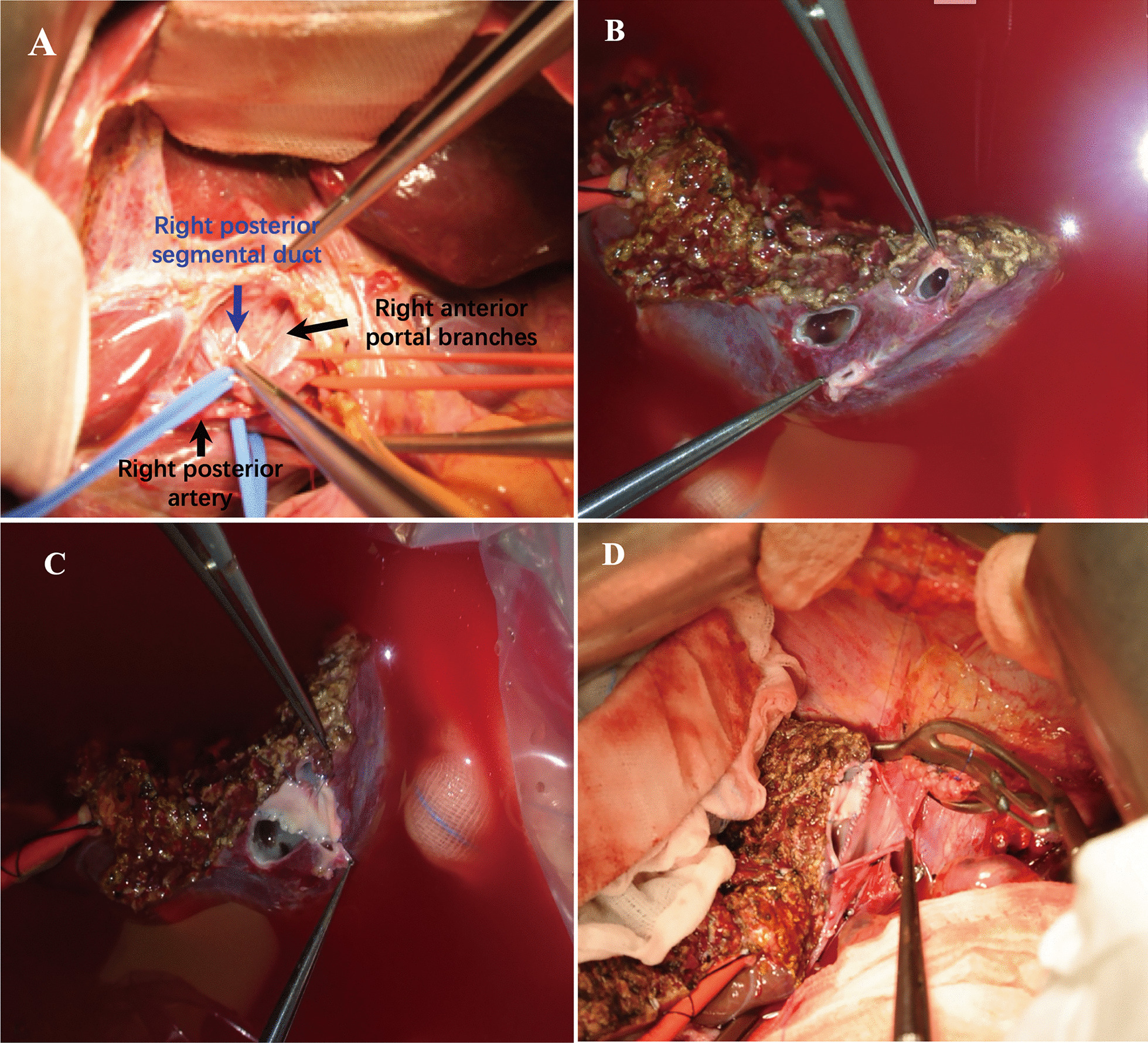
Intraoperative findings during graft-to-recipient reconstructions in case 1. **A** The right posterior bile duct ran on the dorsal side of the portal vein. **B**–**D** 3 orifices of donor liver’s hepatic vein was integrated into one common opening using the autogenous PV patch

Detailed information during transplant surgery was shown in Table 3. The median procured graft weight was 397.5 g (352–461 g) and the median graft-to-recipient weight ratio (GRWR) was 2.38% (1.44–2.80%). In HV reconstruction, middle hepatic veins (MHV) to right hepatic veins (RHV) trunk were applied as anastomotic stomata in 3 recipients to accommodate diameters between grafts and recipients, while the 8-year-old patient used RHV for the reason of her relatively large RHV diameter. Distinguishingly, in case 1, RHV of the donor liver contained 3 orifices. As a result, we performed patch shaping using the autogenous portal vein (PV) patch plastic technique and integrated 3 orifices into one common opening (Fig. 2). The posterior branch of the portal vein (PPV) and the main portal vein (MPV) of the recipient were matched in PV reconstruction process.

**Table3:** Intraoperative characteristics of recipients and their donors

Variable	Case 1	Case 2	Case 3	Case 4
Recipients				
Operation duration (min)	430	395	400	400
Estimated blood loss (ml)	200	100	50	100
Blood transfusion	–	–	–	RBC1U
HV reconstruction (graft-recipient)	RHV-RHV	RHV-trunk (MHV-RHV)	RHV-trunk (MHV-RHV)	RHV-trunk (MHV-RHV)
PV reconstruction	PPV-trunk	PPV-trunk	PPV-trunk	PPV-trunk
HA reconstruction	Single, microsurgery	Single, microsurgery	Single, microsurgery	Single, microsurgery
BD reconstruction	DD	DD	DD	DJ
Donors				
Operation duration (min)	170	175	168	165
Estimated blood loss (ml)	200	100	100	100
Blood transfusion volume (ml)	–	–	–	–
Actual graft weight (g)	405	390	461	352
Actual GRWR (%)	1.44	2.44	2.80	2.32

*PV* portal vein, *HA* hepatic artery, *BD* bile duct, *RHV* right hepatic vein, *MHV* middle hepatic vein, *PPV* posterior portal vein, *DD* bile duct-bile duct, *DJ* bile duct-jejunum

## Discussion

Living donor liver transplantation (LDLT) has now become an efficient treatment to overcome end-stage liver diseases. With the development of global organ shortage, especially in Asian countries, nearly seventy percent of this operations are performed in major Asian transplant centers [9]. However, the additional risk taking to a healthy donor is still controversial. Surgeons need to procure grafts of sufficient volume to fulfill recipients’ liver function requirements and simultaneously ensure donors’ safety. As a result, the preoperative evaluation is usually focused on maintaining the balance of risks between donors and recipients [10]. Moreover, according to previous researches, many factors may affect the actually acquired grafts and functional liver tissues, including the age of donors, the steatotic extent of donor livers and the imaging assessment technology [11]. As a result, the differences between preoperatively estimated and actually harvested graft volume should also cause attention.

In preoperative volumetric evaluation, if the left lateral lobe can satisfy the minimum standard of a recipient’s requirement- 40% of the recipient’s standard liver volume, the left lateral lobe may serve as the first choice in LDLT graft procurement [12]. However, in adult LDLT, left lobes actually cannot provide sufficient graft volume for most patients. Right lobes will be taken into consideration in this situation under the restriction that volume of right lobes are less than 70% of total estimated liver volume in donors [13]. Nevertheless, the procurement of the right lobe graft usually inevitably causes many complications including small remnant volumes, biliary problems and bleeding in the donor. After years of exploration, the right posterior sector (RPS) graft was gradually applied by some centers. The RPS graft is larger than the left lobe in most donors and with the retention of the right anterior sector, the risk is significantly decreased in the donor surgery. Criteria for the application of RPS graft were studied [13]. The RPS graft can be procured in the prerequisite that the volume of RPS is more than 40% of the recipient’s standard liver volume, and the RPS is larger than the left lobe with the caudate lobe. In other words, when the right liver cannot be selected, we choose the larger of the right posterior lobe and the left liver to ensure sufficient graft volume. In order to avoid small-for-size syndrome, sufficient graft volume is an essential factor to ensure the success of the operation in adult living donor liver transplantation.

Some obstacles stand in the way of expanded application of RPS grafts. First, it is difficult to maintain a slight difference between the actual graft volume and estimated graft volume as the parenchymal transection plane is wide. Tiny bias may cause huge deviation during the operation [14]. Second, the vascular stem of RPS branches within the liver parenchyma from second-order structure, which enhances the difficulty in the process of surgical anatomy [15]. In addition, the standard bifurcation of the PV is not suitable for a RPS graft harvesting because the short stump make the reconstruction very difficult. The last worry is concerning the bile duct variation. For instance, dual BDs branching from RPS or posterior BD running dorsally to the PV may make lobectomy very difficult. As a result, the application of RPS grafts still remains scarce all around the world. Special donor anatomy can greatly facilitate the procurement of RPS graft. In preoperative anatomical measurement, the procurement of RPS graft may go through rigorous screening process. The optimal anatomy variation occurs when the right posterior portal vein (PPV) branches from the main PV separately. The right posterior hepatic artery branches outside the liver parenchyma and the right posterior bile duct drains into the common hepatic duct extrahepatically and separately. However, it was reported that only about 1.5% of healthy adults could fulfill all the anatomy requirements mentioned above [16]. Therefore, different centers may have their own expanded anatomical indications according to their surgical experience [17]. In addition to the above-mentioned, the existence and domination area of the IRHV will be also strictly identified during imageological examination to evaluate the demand and difficulty of reconstructions between IRHV and RHV in RPS transplant. Reconstructions will be necessary if the IRHV takes large domination area. This process can be difficult under the condition of unmatched distance between the IRHV and RHV. In our cases, four donors all satisfied the optimal PV anatomy requirements mentioned above, and displayed similar HA and BD anatomical types.

The RPS graft is not only used in adult living donor liver transplantation (LDLT). In pediatric LDLT, especially for some older children who weighed more than 15 kg, the most commonly used left lateral lobe grafts cannot provide sufficient liver tissues for these patients. The criteria in adult LDLT concerning procurement of the RPS graft do not apply to pediatric LDLT. There have been no reports to propose a standard for procurement of the RPS graft in pediatric LDLT up to now. Based on our single-center clinical experience, for older children ranging from 15 to 30 kg, both the right posterior lobe and the left liver can meet the requirements in most cases. Therefore, anatomical factors have become the most important consideration in choosing the right posterior lobe in pediatric LDLT.

Actually, comparing to the left lobe graft, the RPS graft has irreplaceable advantages. For example, it is a contradictory to harvest the left lobe graft with or without the middle hepatic vein (MHV). On the one hand, the left lobe graft without the MHV often has difficulties in the venous return of segment IV. On the other hand, the left lobe graft with the MHV can induce the congestion of segment V and VIII in the remnant donor liver. The right posterior graft can maximize the use of donor liver tissues and reduce the donor’s surgical damage. Furthermore, the RPS graft conforms to the normal physiological structure. The right posterior graft is placed in the right side of the recipient's abdominal cavity, which is in accordance with the physiological position and is not prone to torsion of the bile duct and portal vein in the process of liver regeneration. Thirdly, the type III portal vein has a long right posterior lobe branch, which is more conducive to anastomosis. In addition, for type III portal vein, the left branch of PV belongs to the tertiary branch, and part of the IV segment is supplied by the right anterior branch. As a result, part of the IV segment of the left lobe graft with MHV will lack the PV blood supply and only have the arterial blood supply. Last but not least, the type III portal vein often combines with bile duct variation (the right posterior bile duct often branches independently). In such circumstances, the left liver procurement may easily cause bile duct damage or cut out multiple bile duct openings, which increases the complexity of the operation. In summary, based on our experience, we first proposed the selection criteria for the right posterior graft in LDLT for children weighed more than 15 kg. According to our present study, favorable outcomes after LDLT using RPS grafts could be achieved in pediatric patients through strict volumetric and anatomical assessment of the donor livers.

## Conclusions

**Fig. 3:**
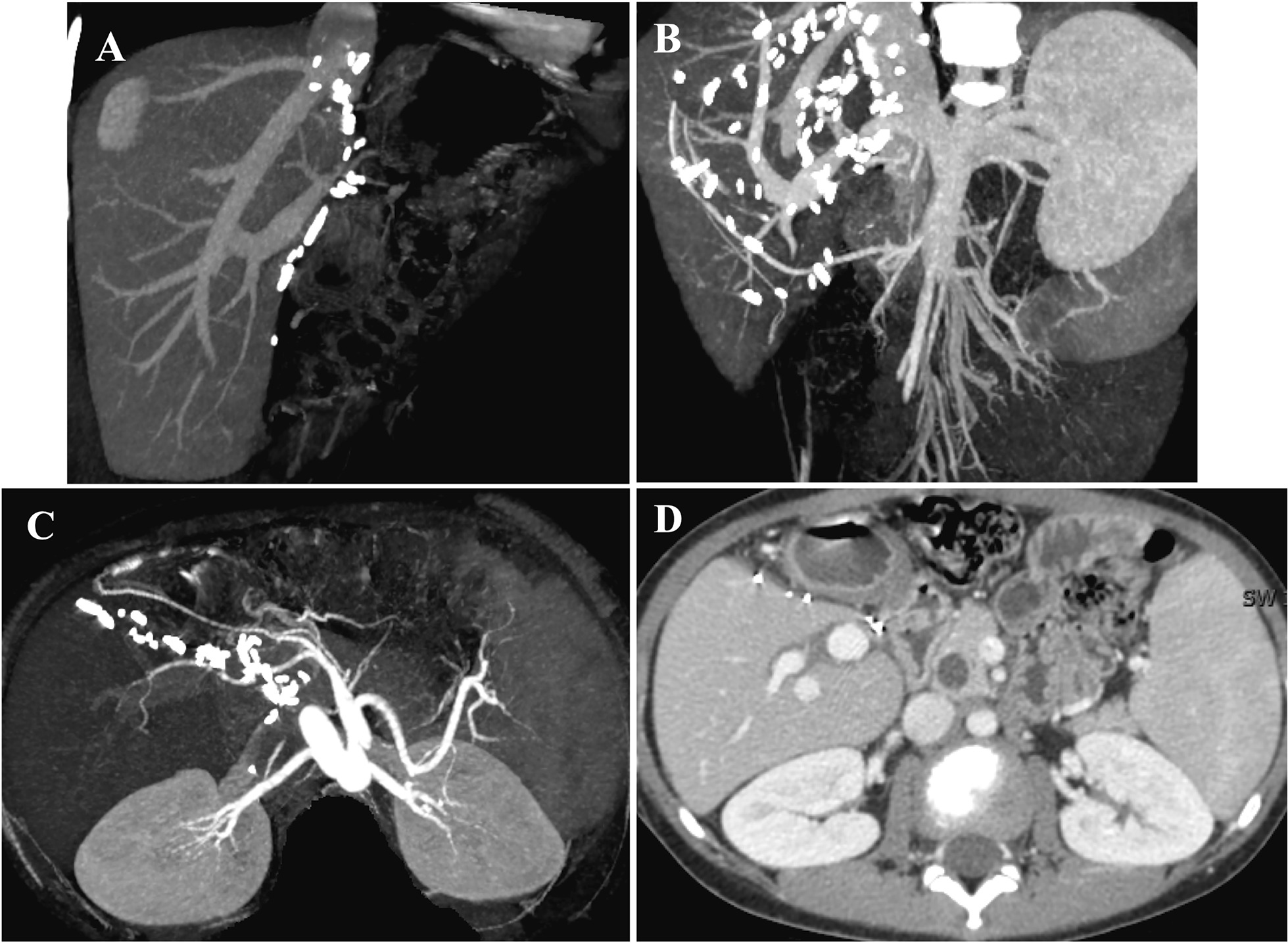
Postoperative imaging of the recipient 13 months after the transplant operation in case 1. **A**, **B** The portal and hepatic vein system. **C** The hepatic artery system. **D** The CT tomography scan showed well graft condition after the transplantation

Four pediatric patients experienced smooth recovery after the surgery. The median length of postoperative hospital stay was 18.5 days (range from 14 to 23 days). The median follow-up duration is 29 months (range from 14 to 64 months) and all recipients are alive with normal graft function. Figure 3 displayed representative CTA images of case1 thirteen months after the transplant operation. The graft was in a well state. As for donors, all donors were discharged within 4 days and it took less than 10 days for the liver function to return to normal level. Postoperative complications occurred in neither donors nor recipients.

## Data Availability

The data and materials used and/or analyzed during the current study are available from the corresponding author on reasonable request.

## Abbreviations

RPS: The right posterior segment
LDLT: Living donor liver transplantation
PFIC: Progressive familial intrahepatic cholestasis
PA: Propionic academia
OTC: Ornithine transcarbamylase
BA: Biliary atresia
CTA: Computer tomography angiography
RHV: Right hepatic veins
GRWR: Graft-to-recipient weight ratio
MRCP: Magnetic Resonance Cholangiopancreatography
